# THE ASSOCIATION BETWEEN OBJECTIVE MEASURES OF INTERMITTENT SEDENTARY BREAKS WITH RECURRENT FALL RISK IN OLDER MEN

**DOI:** 10.1093/geroni/igac059.422

**Published:** 2022-12-20

**Authors:** Lauren Roe, Stephanie Harrison, Peggy Cawthon, Kristine Ensrud, Kelley Gabriel, Deborah Kado, Jane Cauley

**Affiliations:** University of Pittsburgh, Pittsburgh, Pennsylvania, United States; California Pacific Medical Center, Research Institute, San Francisco, California, United States; California Pacific Medical Center Research Institute, San Francisco, California, United States; University of Minnesota Medical School and Minneapolis VA Health Care System, Minneapolis, Minnesota, United States; Department of Epidemiology, The University of Alabama at Birmingham, Birmingham, Alabama, United States; Stanford University School of Medicine, Palo Alto, California, United States; University of Pittsburgh, Pittsburgh, Pennsylvania, United States

## Abstract

**BACKGROUND:**

We assessed the relationship between objectively measured sedentary bout frequency with recurrent falls (2+ falls/year).

**METHODS:**

The sample included 2,918 men aged 79.0±5.1 years attending the Osteoporotic Fractures in Men Study (MrOS) Year 7 (2007-2009) visit. Sedentary bout frequencies were defined as the average daily number of transitions out of 5+ minutes sedentary (<1.5 METS). Falls were self-reported from mailed questionnaires. Generalized Estimating Equations estimated the odds of recurrent falls.

**RESULTS:**

Over four follow-up years, 1,025 (35.1%) men had recurrent falls. Compared to men with <13.6 transitions from sedentary (Quartile (Q)1), the odds of recurrent falls among men with 13.6-<17.0 transitions (Q2), 17.0-<20.4 transitions (Q3), and 20.4-<34.6 transitions (Q4) were 0.82 (95%CI: 0.66-1.01), 0.79 (95%CI: 0.64, 0.99), and 1.01 (95%CI: 0.81, 1.27), respectively, after adjustment.

**CONCLUSIONS:**

A U-shaped association may exist between sedentary bout frequency and recurrent falls risk. The least and most active men are at higher risk.

